# Internal mammary lymph node recurrence: rare but characteristic metastasis site in breast cancer

**DOI:** 10.1186/1471-2407-10-479

**Published:** 2010-09-07

**Authors:** Lei Chen, Yajia Gu, Shiangjiin Leaw, Zhonghua Wang, Peihua Wang, Xichun Hu, Jiayi Chen, Jingsong Lu, Zhimin Shao

**Affiliations:** 1 Department of Medical Oncology, Cancer Hospital of Fudan University, Shanghai Medical College, Shanghai, PR China; 2 Department of Diagnostic Radiology, Cancer Hospital of Fudan University, Shanghai Medical College, Shanghai, PR China; 3 Department of Radiation Oncology, Cancer Hospital of Fudan University, Shanghai Medical College, Shanghai, PR China; 4 Department of Breast Surgery, Cancer Hospital of Fudan University, Shanghai Medical College, Shanghai, PR China

## Abstract

**Background:**

To assess the frequency of IMLN recurrence, its associated risk factors with disease-free interval (DFI) and its predicting factors on overall survival time.

**Methods:**

133 cases of breast cancer IMLN recurrence were identified via the computerized CT reporting system between February 2003 and June 2008, during which chest CT for patients with breast cancer (n = 8867) were performed consecutively at Cancer Hospital, Fudan University, Shanghai, China. Patients' charts were retrieved and patients' characteristics, disease characteristics, and treatments after recurrence were collected for analysis. The frequency was 1.5% (133/8867).

**Results:**

IMLN recurrence was presented as the first metastatic site in 121 (91%) patients while 88 (66.2%) had other concurrent metastases. Typical chest CT images included swelling of the IMLN at the ipsilateral side with local lump and sternal erosion located mostly between the second and third intercostal space. The median disease-free interval (DFI) of IMLN recurrence was 38 months. The independent factors that could delay the IMLN recurrence were small tumor size (HR 0.5 95%CI: 0.4 - 0.8; *p *= 0.002), and positive ER/PR disease (HR 0.6, 95% CI: 0.4 - 0.9; *p *= 0.006). The median survival time after IMLN recurrence was 42 months, with a 5-year survival rate of 30%. Univariate analysis showed four variables significantly influenced the survival time: DFI of IMLN recurrence (p = 0.001), no concurrent distant metastasis (p = 0.024), endocrine therapy for patients with positive ER/PR (p = 0.000), radiotherapy (p = 0.040). The independent factors that reduced the death risk were no concurrent distant metastases (HR: 0.7, 95% CI: 0.4 - 0.9; *p *= 0.031), endocrine therapy for patients with positive ER/PR status (HR: 0.2, 95% CI: 0.1 - 0.5; *p *= 0.001) and palliative radiotherapy (HR: 0.3, 95% CI: 0.1- 0.9; *p *= 0.026).

**Conclusions:**

The risk of IMLN recurrence is low and there are certain characteristics features on CT images. ER/PR status is both a risk factor for DFI of IMLN recurrence and a prognostic factor for overall survival after IMLN recurrence. Patients with only IMLN recurrence and/or local lesion have a good prognosis.

## Background

The significance of internal mammary lymph node (IMLN) as a second lymph node basin in breast cancer where 8%~37% tumors drain to IMLN while 1%~5% of tumor exclusively drain to the IMLN had been recognized as a major prognostic factor historically [1-5]. It was reported that 54% of central and inner quadrant tumors and 18% lateral quadrant tumors were associated with positive IMLN while 44% of cases with positive axillary lymph nodes (ALN) had positive IMLN [6]. However, extended radical mastectomy that included removal of IMLN was abandoned in the 1970s, both as a staging procedure because of a low rate of IMLN metastases in the absence of concomitant ALN metastases, and as a therapeutic procedure because removal of all IMLN did not improve the prognosis. IMLN involvement was found to be 4% to 65% during surgery, 8% to 27% during sentinel node biopsy, and 13% to 37% on lymphoscintigraphy [1-5,7-10]. There was no local control or survival benefit comparing extended radical mastectomy (EM) to radical or modified radical mastectomy while more patients underwent EM experienced surgical trauma and pleura-related complications [11-14]. Yet, renewed interest has been observed for the untreated IMLN following the Early Breast Cancer Trialists' Collaborative Group (EBCTCG) meta-analysis that had established the importance of local-regional control on long- term survival [15].

Following surgery, systemic adjuvant chemotherapy is indicated in selected cases of localized disease based upon prognostic risk factors. All patients with hormone receptor-positive tumors should be considered for adjuvant endocrine therapy lasting for at least 5 years. Radiation of the chest wall and regional lymph nodes is suggested following mastectomy in selected high-risk patients. However, the value of IMLN irradiation without ignoring the risk of cardiac morbidity in the subset of patients with positive IMLN remains unclear presently while the prognostic significance of IMLN recurrences were scarce and controversial. Therefore, we undertake the initiative to review all the patients presented with IMLN recurrence at our institution in order to understand the characteristics associated with IMLN and its predicting influence on the prognosis of breast cancer so that we might have a better understanding in decision making for the most appropriate management for patients who had undergone radical or modified radical mastectomy. The aim of this study was to assess the chance of IMLN recurrence in a single institution, its associated risk factors with disease-free interval (DFI) and its predicting factors on overall survival time. A statistical analysis of a series of 133 breast cancer patients with IMLN recurrences is presented.

## Methods

IMLN recurrence in our study was defined as a soft nodule with a diameter of 1 cm or larger adjacent to the internal mammary vessels within the first through sixth parasternal anterior intercostal space on spiral chest CT scan and/or confirmed by cytology. Surgical management usually performed at our center for patients with primary breast cancer included modified and radical mastectomy. Adjuvant chemotherapy for four to six cycles of cyclophosphamide, methotrexate, and 5-fluorouracil (CMF), cyclophosphamide, epirubicin, and 5-fluorouracil (CEF) and CEF followed by docetaxel are usually given for patients with node-positive disease or at high risk despite having node-negative disease. Adjuvant radiotherapy at a dose of 45-50 Gy delivered to fields including chest wall and supraclavicular area with a 10-Gy boost to tumor bed using targeted fields of electrons are recommended for postmastectomy patients with primary tumor size greater than 5 cm, 4 or more positive axillary lymph nodes, or positive pathologic margins. Adjuvant endocrine therapy was given for patients with ER/PR positive tumors while trastuzumab was not available for patients with HER-2 positive disease then. Patient was followed-up every 3 months after primary surgery with documented physical examination, every 6 months for ultrasonic examination, every 12 months for conventional CT-scan further spiral chest CT scan if indicated.

To identify patients with IMLN recurrence, we reviewed all chest spiral CT (computed tomographic) reports covering a 5-year span from Feb 2003 through June 2008 when CT reports were recorded by computer at Cancer Hospital of Fudan University, Shanghai, China, which served a population of almost half million. All CT examinations were performed using a 40-row helical CT scanner operated at 120 kV and 100 mAs, with a maximized 45 × 45 cm field of view, a 512 × 512 matrix, and the table speed of 1.53 mm/0.5 seconds (Somatom-40-row, Siemens Medical, Germany). CT images were obtained using consecutive 5 mm-thick CT scanning from the supraclavicular region to the top of diaphragm with breath holding. Each CT scan which reported internal mammary lymph node enlargement was reviewed again by two radiologists for the confirmation of clinically apparent IMLN recurrence.

The frequency of IMLN recurrence in this series was 1.5% (133/8867) after radical (n = 59) or modified radical mastectomy (n = 74). The primary surgery time was between August 1988 and June 2007, the median surgery time was Mar 2003. Patients who received primary extended radical mastectomy were excluded from this study. IMLN status was not evaluated by sentinel LN biopsy.

The median age at surgery was 57 years, with 78 (58.6%) patients being postmenopausal. The median primary tumor size was 2.5 cm (range: 1-7 cm), among which 31.6% (n = 42) was ≤ 2 cm, 63.2% (n = 84) was between 2 cm to 5 cm, and 5.2% (n = 7) > 5 cm. Number of ALN involvement was negative in 46.6% (n = 62) patients, 1~3 in 32.3% patients (n = 43), and ≥4 in 21.1% patients (n = 28). According to 6^th ^AJCC staging manual, 17 (12.8%) patients had stage I, 89 (64.4%) had stage II, and 29 (21.8%) had stage III disease. 78 (58.6%) patients were ER/PR positive. 29 (21.8%) had HER-2 positive disease (HER-2 positive was defined as +++ by IHC method or FISH positive). The most common histological type was invasive ductal carcinoma, with 46% of the tumors located at the areola and inner quadrant area.

After primary surgery, 124 (93.2%) patients received adjuvant chemotherapy. Only 15.8% (21/133) received adjuvant radiotherapy due to worrisome of the toxicity of radiotherapy. All patients with ER/PR positive tumors received adjuvant endocrine therapy (73 of 78 patients received tamoxifen 20 mg daily, another 5 patients received aromatase inhibitor). The median follow-up time was 52 months (range: 10-246 months). All patients' characteristics were listed in Table 1.

**Table 1:** Patient Characteristics

	Patients	
	
Characteristic	N	%
*Age (years)*		
Median (range)	47 (23-72)	
*Menopausal stastus*		
Premenopausal	55	41.4
Postmenopausal	78	58.6
*Histologic type*		
Invasive ductal carcinoma	125	94.0
Invasive lobular carcinoma	5	3.8
Medullary carcinoma	3	2.2
*Primary tumor location*		
Areola and inner area	61	45.9
Outer area	72	54.1
*Primary tumor size (cm)*		
Median (range)	2.5 (1-7)	
≤2	42	31.6
>2	91	68.4
*Anxillary lymph node (ALN)*		
negative	62	46.6
positive	71	53.4
*TNM stage*		
I - II	104	78.2
III	29	21.8
*ER/PR status*		
Positive	78	58.6
Negative	55	41.4
*HER-2 status*		
Positive^&^	29	19.5
Negative	104	80.5
*Adjuvant chemotherapy*		
Yes	124	93.2
No	9	6.8
*Adjuvant radiotherapy #*		
Yes	21	15.8
No	112	48.2

^&^HER-2 positive was defined as +++ by IHC method and/or FISH positive.

#Adjuvant radiotherapy field included chest wall and supraclavicular area, and 10-Gy boost was delivered to the tumor bed

90% patients were followed as planned while 10% present with recurrent disease at a more serious condition due to poor compliance. All 133 patients were evaluated for distant metastases using abdominal CT/MRI and bone scan after the diagnosis of IMLN recurrence. The types of therapy right after IMLN recurrence were recorded. Endocrine therapy was most commonly used for patients with ER/PR positive disease or at low risk of rapidly progressive disease. Patient with ER and PR negative, life-threaten condition or multiple metastatic sites often received chemotherapy. Radiotherapy was given either as initial or palliative treatment based on patients' disease presentation.

DFI of IMLN recurrence was calculated from the primary surgery to IMLN recurrence. The overall survival time was calculated from the IMLN recurrence until death or last follow-up. Actuarial curves were compared by the two-tailed log-rank test and difference of p ≤ 0.05 was considered significant. The independent prognostic significance of variables on the events and survival, proved to be significant factor in univariate analysis, was tested in proportional hazards regression models described by Cox. Survival analysis was carried out using life-table and Kaplan-Meier method. The estimates of the models are given as hazard ratio (HR) with 95% confidence intervals (95% CI).

Written informed consent was obtained from all patients at the time of admission. The study had been approved by the Fudan University Cancer Hospital Ethic Committee for Clinical Investigation. Patients' charts were retrieved and patients' characteristics, disease characteristics, and initial treatments after IMLN recurrence and patients' survival were collected for analysis.

## Results

### Characteristics of IMLN recurrences

#### Clinical characteristics

Among the 133 patients, IMLN recurrence was presented as the first metastatic site in 121 (91.0%) patients, only 12 had other sites of metastases prior to IMLN recurrence. 88 (66.2%) patients had other concurrent metastasis sites, 45 patients had isolated IMLN recurrence, of whom 23 developed distant metastases later. There were 48 cases (36.1%) with ipsilateral visible soft tissue mass beside the sternum, among which cytology examination had been presented for 33 patients, all confirmed recurrence. There were 20 (15.0%) cases with localized skin involvement with painful presentation.

#### Imaging of chest spiral CT scan

**Figure 1:**
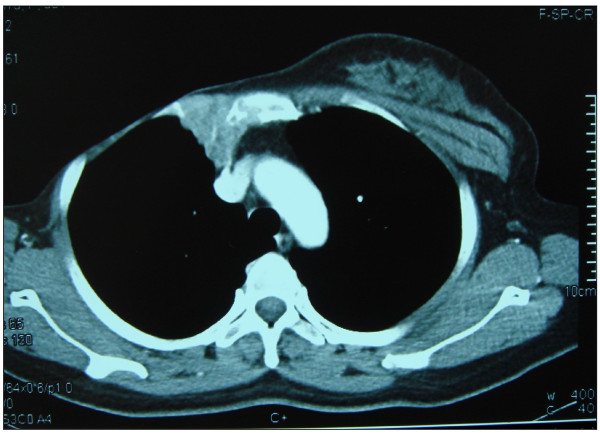
**CT image demonstrated internal mammary node forming a mass with a diameter of 3 cm at a level of the second right anterior intercostal space accompany by sternal erosion**.

The typical CT-scan presentation was enlargement and swelling of IMLN located on the ipsilateral side with the formation of a local lump and sternal erosion (Figure 1). Median enlarged nodal size was 2.5 cm (range: 1.0-9.0 cm). The majority of the cases presented with lymphadenopathy traversing one to two anterior intercostal spaces, being 48.1% and, 33.9%, respectively. Most nodal enlargement was located at the second and third intercoastal space, being 67.7% and 19.5%, respectively. Isolated lymphadenopathy in the fifth spaces was not observed. About half of the cases with IMLN recurrence occurred at multiple levels and were most common in the second or third intercoastal spaces. Sternal erosion occurs in 64.7% patients. The characteristics of the imaging findings were presented in Table 2.

**Table 2:** Characteristics of IMLN Recurrence

	N	%
*Clinical*		
*As first site of metastasis*		
Yes	121	91
No	12	9
*Concurrent other sites metastasis*		
Yes	88	66.2
No	45	33.8
*Visible soft tissue mass beside the sternum*		
Yes	42	31.6
No	91	68.4
*Local pain and skin involvement*		
Yes	20	15.0
No	113	85.0
*Imaging of CT scan*		
*Size of IMLN recurrence (cm)*		
Median (range)	2.5 (1-9)	
*Anterior intercostal space*		
One	64	48.1
Two	45	33.9
Three	19	14.3
Four	5	3.7
*Dominant location anterior intercostal space*		
First	15	11.3
Second	90	67.7
Third	26	19.5
Forth	2	1.5
*Sternal erosion*		
Yes	86	64.7
No	47	35.3

### Risk Factor of DFI for IMLN recurrence

The median DFI of IMLN recurrence was 38 months (range: 4-241 months). Tumor size, ER/PR status, adjuvant radiotherapy for T > 5 cm were variables that significantly influenced the DFI of IMLN recurrence (Table 3). The DFI for patients with primary tumor size ≤ 2 cm and > 2 cm was 49 and 33 months, respectively (p = 0.021). The DFI for patients with positive and negative ER/PR was 50 and 32 months, respectively (p = 0.020). For patients with T > 5 cm, DFI was 56 months and 21 months for those with or without adjuvant radiotherapy, respectively p = 0.018. All the following tested variables had no influence on the DFI of IMLN recurrence: menopausal status, primary tumor location, number of ALN involvement, post-surgery TNM stage, HER-2 status, adjuvant chemotherapy, adjuvant radiotherapy for ≥ 4 positive ALN.

**Table 3:** Univariate and multivariate analysis of DFI for IMLN recurrence

Factors	N	Univariate analysis		Multivariate analysis		
		
		Median (m)	*p*-value	HR	95% CI	*p*-value
*Menopausal stastus*			0.773	NS		
Premenopausal	55	33				
Postmenopausal	78	42				
*Primary tumor location*			0.837	NS		
Areola and inner area	61	38				
Outer area	72	40				
*Tumor size(cm)*			0.008*	0.5	0.4- 0.8	0.002*
≤2	42	49				
>2	91	33				
*Axillary lymph node*			0.070	NS		
negative	62	43				
positive	71	33				
*TNM stage*			0.390	NS		
I - II	104	38				
III	29	29				
*ER/PR status*			0.020*	0.6	0.4- 0.9	0.006*
Positive	78	50				
Negative	55	32				
*HER-2 status*			0.193	NS		
Positive	29	26				
Negative	104	42				
*Adjuvant chemotherapy*			0.113	NS		
Yes	124	36				
No	9	41				
*Adjuvant radiotherapy for T *>*5 cm*			0.018*	0.9	0.6- 1.3	0.463
Yes	3	56				
No	4	21				
*Adjuvant radiotherapy for ALN *≥*4*			0.949	NS		
Yes	20	29				
No	8	26				

* *p *< 0.05

Three factors were tested in multivariate analysis: tumor size, ER/PR status, adjuvant radiotherapy for T >5 cm. The result showed that the DFI of IMLN recurrence was independently influenced by tumor size (HR 0.5, 95% CI: 0.4 - 0.8; p = 0.002), and ER/PR status (HR 0.6, 95% CI: 0.4 - 0.9; p = 0.006) (Table 3).

### Prognostic factor after IMLN recurrence

The median survival time after IMLN recurrence was 42 months, and the 5-year survival rate was 30% for all patients. The 5-year survival of patients without concurrent other sites metastasis was 43%. The results of univariate analysis of the tested prognostic variables are shown in Table 4. After univariate analysis, four predicting variables significantly influenced the survival time. DFI of IMLN recurrence (< 2 years vs ≥ 2 years, p = 0.001), concurrent metastasis (none and local vs distant metastases, p = 0.024), endocrine therapy for patients with positive ER/PR (yes vs no, p = 0.000), radiotherapy (yes vs no, p = 0.040). Whether IMLN presented as first or subsequent metastatic site, the size of recurrent IMLN and the type of first-line chemotherapy had no influence on the overall survival time. The median overall survival time for patients with isolated IMLN recurrence was 63 months, patients with concurrent local metastasis 51 months (p = 0.432). The median overall survival time when both isolated IMLN and with local metastases was combined was 61 months, while that of concurrent bone or visceral metastasis (lung or liver or brain, etc) was 32 months. Univariate analysis also showed that the presence of concurrent distant metastases (p = 0.010) predicted the survival time (Table 4).

**Table 4:** Univariate and multivariate analysis of survival time after IMLN recurrence

Factors	N	Univariate analysis		Multivariate analysis		
		
		Median (m)	*p*-value	HR	95%CI	*p*-value
*As first site of metastasis*			0.407	NS		
Yes	121	34				
No	12	64				
*Size of IMLN*			0.984	NS		
≤4 cm	116	51				
>4 cm	17	38				
*DFI of IMLN recurrence*			0.001*	1.4	0.7- 2.9	0.342
<2 y	44	31				
≥2 y	89	63				
*Concurrent metastasis*			0.024*	0.7	0.4- 0.9	0.031*
No and local	73	61				
Visceral or bone	60	32				
*Endocrine therpay*			0.000*	0.2	0.1- 0.5	0.001*
Yes	54	78				
No	24	26				
*Chemotherapy*			0.078	NS		
Yes	72	34				
No	61	62				
*Radiotherapy*^*&*^			0.04*	0.3	0.1- 0.9	0.026*
Yes	59	63				
No	74	32				

^&^Radiotherapy included the patients who received RT as initial (7 cases) and palliative (52 cases) treatment

*p < 0.05

The four factors proved to be significant in univariate analysis were tested by multivariate analysis (shown in Table 4). The following independent factors that reduced the risk of death were no concurrent distant metastases (HR: 0.7, 95% CI: 0.4- 0.9; *p *= 0.031), presence of endocrine therapy for patients with positive ER/PR (HR: 0.2, 95% CI: 0.1 - 0.5; *p *= 0.001), presence of sequential radiotherapy delivered to the IMLN area (HR: 0.3, 95% CI: 0.1- 0.9; *p *= 0.026).

## Discussion

This is the largest report of IMLN recurrences up to date. The frequency of IMLN recurrence was rare [16,17], 1.5% in our series, and even rarer among patients with other cancer types. But it was characteristic recurrence site for the patients with breast cancer, only two patients with other solid tumors, both male, were noted to have IMLN metastases on CT scan in our institution, one suffered from malignant melanoma, and the other rhabdomyosarcoma. Two independent factors were found to delay IMLN recurrence among patients with breast cancer: small tumor size, and positive ER and PR status. Three independent factors were identified to increase death risk: absence of endocrine therapy for patients with positive ER/PR status, absence of palliative radiotherapy and concurrent distant metastases.

The diagnosis of IMLN recurrence was based on the clinical presentation and chest CT-scan [18]. Clinical symptoms may include broad base parasternal swelling with concomitant pain or skin involvement. Some patients with IMLN recurrence were discovered on a chest CT-scan during routine follow-up. The typical CT-scan presentation was swelling of IMLN, usually located at the ipsilateral side of the treated breast, with a local lump and sternal erosion. About half of the IMLN recurrence occured at multiple levels and were most commonly seen in the second or third intercoastal space. Most IMLN recurrence, 66.2% in this series, presented concurrently with other metastases sites, or were followed by visceral metastasis similar to those of other reports [19]. Therefore, once IMLN recurrence was detected, a thorough re-staging workup was necessary to evaluate the extent of the disease status.

Recurrence in the IMLN is rare, despite the fact that these nodes are the second LN drainage basin of breast cancer and are left untreated after surgery in most patients. Tumor location, positive ALN, younger age, and larger tumor size (>5 cm) had been reported as high risk factors of tumor cells drainage to IMLN [8], but the risk factors of IMLN recurrence had not been clarified. Tumor size has been reported as a risk factor not only for IMLN drainage but also for local recurrence [8,19,20]. Harris et al reported T2 tumor size (*P *= 0.004), and Stage II disease (*P *= 0.017) were associated significantly with any regional lymph node recurrence (including ALN, IMLN, supraclavicular lymph node). Bijker et al reported the significant independent factors were tumor size > 5 cm (measured by pathology) (p = 0.0002). Conflicting results have been reported regarding the influence of ALN status on regional recurrence [19,20]. Since all patients in our present study had developed IMLN recurrence, factors that could influence the DFI of IMLN recurrence instead of risk factors for IMLN recurrence were evaluated. We concluded that tumor size, not ALN status, could influence the interval of IMLN recurrence.

Several studies reported that adjuvant treatment modalities did not influence the risk of local recurrence after breast-conserving surgery for early-stage disease [21-23]. However, adjuvant hormonal therapy was observed to delay IMLN recurrence for patients with ER/PR positive disease in our study. We postulated that different surgical methods and stage at presentation in our series may contribute to the different conclusion. The finding in our series that adjuvant radiotherapy could significant delay the IMLN recurrence for patients with tumor size > 5 cm, paralleled other studies on postmastectomy radiation delivered to fields including IMLN, supraclavicular, axillary, and chest wall, which demonstrated significant decreased risk of locoregional recurrence in high-risk breast cancer patients [24,25]. The significance of adjuvant chemotherapy for delayed IMLN recurrence could not be demonstrated in our study due to the fact that the majority of patients included in this analysis received adjuvant chemotherapy per NCCN guidelines.

The 5-year survival rate of the 133 patients was 30% while that for those with isolated IMLN recurrence was 43%. The results is similar to the findings of other studies on isolated local recurrence (IRL) of breast cancer, ranging from 39-84%, IRL defined as new breast tumor, chest wall recurrence, overlying skin recurrence, supraclavicular recurrence [21,26-29]. Since isolated IMLN recurrence, longer DFI to IMLN recurrence, endocrine therapy and radiotherapy were four favorable prognostic factors for overall survival time, we propose that IMLN recurrence should be included in the definition for ILR.

Time to ILR, the most frequently reported prognostic factor for survival after local recurrence post breast-conserving surgery was also reported in our study for IMLN recurrence after radical or modified radical breast cancer surgery [28-30]. Endocrine therapy and radiotherapy could prolong the survival time dramatically but the benefit of chemotherapy was not observed in this study. This may be due to the fact that patients indicated for initial chemotherapy had a more advanced disease condition accompanying IMLN recurrence. However, due to the rarity of IMLN recurrence, it seemed difficult to initiate a prospective clinical trial to evaluate the influence of systemic treatment and radiotherapy on the survival.

The 2008 National Comprehensive Cancer Network Clinical Practice Guidelines recommend consideration of radiation therapy to internal mammary nodes for patients with node-positive breast cancer after mastectomy or breast-conserving surgery, noting "substantial controversy" on this topic. But as isolated IMLN recurrence is rare, it is unlikely that parasternal irradiation will result in a significant reduction of the risk of clinically apparent IMLN recurrence, based on the negligible risk of IMLN recurrence without adjuvant radiotherapy. Thus, we strongly advocate that if IMLN is clinical or pathological negative, it should be left untreated regardless of the status of axillary nodes.

## Conclusions

In conclusion, our data show that the risk of IMLN recurrence is low and there are certain characteristics features on CT images. ER/PR status is both a risk factor for DFI of IMLN recurrence and a prognostic factor for overall survival after IMLN recurrence. Patients with only IMLN recurrence and/or local lesion have a good prognosis.

## Competing interests

The authors, their immediate families of this paper have no potential conflict of interest to disclose.

## Authors' contributions

LC participated in acquisition of data, analysis and interpretation of data, and drafting of the manuscript. YG and PW confirmed chest spiral CT. SL revised the manuscript. XH, JC, JL and ZS participated in acquisition of data. ZW conceived of the study, participated in its design and coordination and revised the final manuscript. All authors have read and approved the final manuscript.

## Pre-publication history

The pre-publication history for this paper can be accessed here:

http://www.biomedcentral.com/1471-2407/10/479/prepub
